# Service-oriented reallocation and value-added intensity in China’s sport industry, 2018–2024: A longitudinal accounting analysis

**DOI:** 10.1371/journal.pone.0354049

**Published:** 2026-10-08

**Authors:** Qiuyuan Peng, Minglin Wu, Qiang Feng

**Affiliations:** 1 School of Physical Education, Yunnan Normal University, Kunming, Yunnan, China; 2 School of Management, Qujing Normal University, Qujing, Yunnan, China; 3 School of Physical Education, Southwest Forestry University, Kunming, Yunnan, China; University of Naples Federico II: Universita degli Studi di Napoli Federico II, ITALY

## Abstract

China’s sport industry expanded while its composition shifted toward services during 2018–2024, but both the accounting mechanisms behind this change and the robustness of the pattern to the COVID-19 disruption and relative-price movements require careful qualification. We analyze annual national sport-industry accounts published by the National Bureau of Statistics of China and the General Administration of Sport. The analysis combines value-added shares, value-added intensity, a descriptive service-to-manufacturing value-added ratio, shift-share decompositions, alternative time windows, a proxy-based price sensitivity analysis, and crisis–recovery indicators. The service share of sport-industry value added increased from 64.8% in 2018 to 72.0% in 2024, while the manufacturing share declined from 33.7% to 26.5%. Aggregate value-added intensity rose from 37.9% to 41.7%. For 2018–2024, the decomposition assigns +2.50 percentage points to changes in category output weights, + 1.44 points to within-category intensity changes, and −0.13 points to their interaction. The between-category contribution remained positive in all alternative multi-year windows, although it was not always larger than the within-category contribution. Under the specified price-proxy scenario, the between-category contribution remained positive. Co-located services fell by 16.2% in value added in 2020 and rebounded by 24.6% in 2021, ending 4.4% above their 2019 level; education/training and media/information services did not contract in 2020. The published accounts therefore document a service-oriented reallocation over the observed period, alongside substantial post-shock within-category change and heterogeneous exposure across service activities. Because the series uses current-price data and crosses a 2023 census-benchmark break, the results should be interpreted as descriptive accounting patterns rather than causal estimates of productivity, policy effects, or pandemic mechanisms.

## Introduction

Sport economies are not composed only of manufactured goods. National sport satellite accounts increasingly identify services, participation, venues, media, information and other experience-based activities as important components of sport-related value added. Recent accounts for the United Kingdom and the European Union, together with input–output work in Japan, illustrate both the economic importance of sport services and the methodological effort required to define and compare sport across national accounting systems [1–3]. At the same time, sport-management research has largely conceptualized value at the level of organizations, events, consumers and service ecosystems. The Sport Value Framework, recent eSports research and studies of sport live-streaming platforms emphasize that value is produced through interactions among multiple actors rather than residing only in a physical product [4–6]. These perspectives motivate attention to the composition of national sport economies, but macroeconomic accounting ratios should not be treated as direct measures of consumer value co-creation.

A broader economic literature provides a second reason to study composition. Classical accounts of structural development describe shifts in activity from primary and manufacturing production toward services as economies develop [7–10]. Research on servitization likewise examines movement from stand-alone products toward service-rich offerings and business models [11,12], while service-dominant logic treats service as a fundamental basis of exchange [13–15]. More recent work on digital servitization shows how digital capabilities can alter the organization and delivery of services [16]. These traditions suggest that a growing service share may accompany changes in how an industry generates value added. Yet an increase in an industry-wide value-added ratio can arise mechanically if output shifts toward categories that already have higher value-added intensity, even when no category changes internally. Distinguishing reallocation from within-category changes is therefore necessary before interpreting an aggregate rise.

China provides a useful accounting case because its official sport-industry system reports annual gross output and value added for a large manufacturing base, a broad service category and facility construction. A recent PLOS ONE study used grey relational and forecasting methods to examine China’s sport-industry structure [17], but the contribution of compositional reallocation to aggregate value-added intensity, the robustness of that contribution across the COVID-19 period, and the heterogeneous recovery of service activities remain less directly documented. The 2018–2024 window is informative but short: it contains the abrupt 2020 contraction, the 2021 rebound, and a benchmark revision associated with the Fifth National Economic Census. Sport research during the pandemic also documents major disruption to events and networks and the rapid use of digital technologies by sport organizations and small sport businesses [18–21]. These features make it important to separate descriptive accounting patterns from causal claims.

This study therefore asks three questions. First, how did the composition and value-added intensity of China’s sport industry change from 2018 to 2024? Second, how much of the observed change in aggregate value-added intensity is associated with shifts in category output weights versus changes within categories, and how stable is that decomposition across alternative time windows and a proxy-based price sensitivity analysis? Third, how did sport service activities with different dependence on physical co-presence differ during the 2020 shock and 2021 recovery? We address these questions with a reproducible accounting analysis of official aggregate data. The aim is to characterize the observed reallocation and recovery pattern while keeping the interpretation proportionate to a short, current-price, non-causal time series. Here, ‘longitudinal’ denotes a seven-year annual aggregate accounting series rather than a causal longitudinal or individual-level panel design. The study does not introduce a new accounting metric or decomposition method; its incremental contribution is the transparent and reproducible application of established accounting and shift-share techniques to an updated national sport-industry series, separating between-category reallocation from within-category VAI change and examining the stability of that distinction across alternative time windows, a relative-price proxy scenario, and the 2020–2021 shock.

## Materials and methods

### Data source and statistical classification

The study uses annual official accounts for China’s sport industry for 2018–2024. The accounts are published by the National Bureau of Statistics of China (NBS) together with the General Administration of Sport of China and follow the Statistical Classification of Sports Industry (2019) [22]. The classification defines the sport industry as production activities that provide sport goods, sport services and sport-related products. At the first tier, the accounts distinguish sport services, manufacture of sporting goods and related products, and construction of sport facilities. The service category is further disaggregated into management activities; competition and performance; fitness and leisure; venue and facility management; agency, advertising, exhibition and design; education and training; media and information; sporting-goods sales, rental and trade agency; and other sport services. The seven annual releases provide gross output and value added at current prices and report composition shares [23–29].

All published values used in the analysis were re-audited against the corresponding official tables. Source URLs, original units and row-level values are recorded in S1 Dataset. Official headline totals are retained for aggregate descriptive statistics, whereas the shift-share decomposition uses the published first-tier category values so that category weights and intensities come from the same set of components. Small discrepancies between headline totals and the sum of rounded category values are preserved rather than mechanically adjusted; for example, the official 2018 total value added is RMB 1,007.8 billion while rounded first-tier components sum to RMB 1,007.9 billion, and the official 2020 total gross output is RMB 2,737.2 billion while rounded components sum to RMB 2,737.1 billion. The 2023 release was benchmarked using the Fifth National Economic Census [28]. That release states that the previous-year comparison data were historically revised on the basis of the census. The present 2018–2022 observations, however, retain the values from the contemporaneous annual releases because the 2023 announcement does not provide a complete backward-revised category panel with both gross output and value added for all pre-2023 years. Accordingly, comparisons that cross the 2022–2023 boundary are not fully homogeneous in benchmark basis, and part of the apparent change may reflect statistical rebenchmarking rather than economic reallocation alone.

The data are aggregate, publicly available economic statistics and contain no human participants, animals or identifiable personal information. Institutional ethics review was therefore not applicable.

### Accounting measures

For category i in year t, value-added intensity (VAI) is defined as VAI(i,t) = V(i,t)/ G(i,t), where V is value added and G is gross output. In national accounting, value added represents gross output less intermediate consumption. VAI is therefore an accounting ratio describing the proportion of gross output represented by value added. It is not interpreted here as labor productivity, total factor productivity, profitability, technical efficiency or welfare. Aggregate VAI is a gross-output-weighted average of category-specific VAI and consequently depends on both category intensities and the composition of gross output.

To summarize the relative scale of service and manufacturing value added, we report a service-to-manufacturing value-added ratio (SMVAR), defined as SMVAR(t) = V(services,t)/ V(manufacturing,t). This is a descriptive composition statistic rather than a validated performance index. Values above one indicate that sport services account for more value added than sporting-goods manufacturing. Because the ratio is sensitive to relative prices and to the choice of denominator, it is interpreted together with value-added shares and is included in the price sensitivity analysis.

### Shift-share decomposition and temporal robustness

To separate compositional and within-category contributions, the change in aggregate VAI between a base year 0 and an end year 1 is decomposed across the three first-tier categories. Let w denote a category’s share of gross output and v its VAI. The decomposition is:


$$\begin{array}{c} \Delta VAI\;=\;\Sigma_{\text{i}}(w_{\text{i1}}\;-w_{\text{i0}})v_{\text{i0}}\;+\;\Sigma_{\text{i}}w_{\text{i0}}(v_{\text{i1}}\;-\;v_{\text{i0}})\;+\;\Sigma_{\text{i}}(w_{\text{i1}}\;-\;w_{\text{i0}})(v_{\text{i1}}\;-\;v_{\text{i0}}) \end{array}$$

The first term is the between-category contribution: the change associated with shifts in gross-output weights evaluated at initial category intensities. The second is the within-category contribution: the change associated with category-specific VAI evaluated at initial weights. The third is an interaction term. Because the series contains only seven annual observations and spans the pandemic shock, the 2018–2024 endpoint decomposition is supplemented with alternative windows (2018–2022, 2018–2023, 2019–2024, 2020–2024 and 2021–2024), each adjacent-year decomposition, and a full-period calculation excluding facility construction. These checks assess whether the sign and relative magnitude of the components depend on a particular endpoint.

### Proxy-based price sensitivity

The official sport-industry accounts are reported at current prices, and a complete set of sport-specific output and value-added deflators is not available for the study period. Current-price results therefore remain the primary analysis. To assess whether differential price movements between services and manufacturing could plausibly overturn the compositional finding, we conduct a deliberately limited proxy-based sensitivity analysis. Sport services are deflated using the national annual CPI service-price index reported by the NBS [30]. Sporting-goods manufacturing is deflated using the national producer-price index for the broader category ‘manufacture of cultural, educational, arts and crafts, sports and entertainment products’, reported in the China Statistical Yearbook series [31]. Cumulative indices are rebased to 2018 = 1; exact annual index values and source links are provided in S1 Dataset.

Gross output and value added within a first-tier category are divided by the same category-specific price proxy. This leaves category-specific VAI unchanged by construction but allows differential price movements to change relative real-weight proxies, aggregate proxy-weighted VAI and SMVAR. Facility construction is held at its current-price value in the primary proxy exercise because national fixed-asset-investment price statistics were discontinued from 2020 [32]. We therefore also repeat the full-period decomposition after excluding construction. These calculations are sensitivity checks rather than official constant-price national accounts: CPI service prices and the broader manufacturing PPI do not provide the product-level double deflation required for official volume measures. Accordingly, the exercise is best interpreted as a sensitivity test of relative category weights under specified price proxies, not as a full inflation-adjusted test of category-specific VAI or as evidence that price effects have been eliminated.

### Crisis–recovery classification and indicators

The crisis–recovery analysis uses 2019 as the pre-shock reference, 2020 as the shock year and 2021 as the first recovery year. To reduce classification discretion, exposure types are defined operationally before comparison. ‘Co-located’ services are those whose core delivery normally requires simultaneous physical attendance at a sport venue, activity or live event: competition and performance, fitness and leisure, and venue and facility management. ‘Knowledge’ refers to education and training. ‘Digital’ refers to media and information services. ‘Mixed’ includes management; agency, advertising, exhibition and design; sporting-goods sales, rental and trade agency; and other sport services because their official category labels combine activities with heterogeneous delivery modes. Manufacturing is retained as a non-service comparator.

For each sub-sector and exposure group, we calculate the 2020 percentage change from 2019, the proportion of the 2019 value-added level retained in 2020, the 2021 percentage rebound from 2020, and the 2021 percentage difference from the 2019 baseline. For categories that contracted in 2020, a recovery ratio is calculated as (VA2021 − VA2020)/ (VA2019 − VA2020); values above one indicate that the 2021 current-price value-added level exceeded the 2019 level. Classification sensitivity reassigns agency/advertising/exhibition/design to the co-located group and, separately, treats education/training as mixed.

### Computational reproducibility and analytical scope

All analyses are deterministic transformations of published aggregate accounts; no stochastic estimation, hypothesis test, p value or confidence interval is used. The complete source log, raw first-tier and sub-sector data, classification rules, formulas, decomposition windows, price-proxy inputs and derived results are provided in S1 Dataset. S2 File contains Python code that reconstructs the annual measures, shift-share decompositions, price sensitivity, crisis–recovery indicators and Figs 1–3 from S1 Dataset. Calculations were reproduced using Python 3.13.5 and pandas 2.2.3. The design supports descriptive characterization of accounting relationships, not causal inference about servitization, government policy, digitalization, productivity or pandemic restrictions. Throughout the manuscript, ‘longitudinal’ refers only to this seven-year annual aggregate accounting series.

## Results

### Aggregate expansion and value-added intensity

China’s sport-industry gross output increased from RMB 2,657.9 billion in 2018 to RMB 3,842.1 billion in 2024, while value added increased from RMB 1,007.8 billion to RMB 1,603.3 billion (Table 1). The corresponding nominal compound annual growth rate of value added was 8.0%. The series was not monotonic: total value added fell by 4.6% in 2020 and rose by 14.1% in 2021. Aggregate VAI increased from 37.9% in 2018 to 41.7% in 2024. Because these are current-price accounts, the growth rates combine quantity and price changes; the ratio measures are interpreted as accounting composition rather than real productivity growth.

**Table 1:** Gross output, value added and aggregate value-added intensity of China’s sport industry, 2018–2024.

Measure	2018	2019	2020	2021	2022	2023	2024
Gross output (RMB billion)	2,657.9	2,948.3	2,737.2	3,117.5	3,300.8	3,674.1	3,842.1
Value added (RMB billion)	1,007.8	1,124.8	1,073.5	1,224.5	1,309.2	1,491.5	1,603.3
Aggregate VAI (%)	37.9	38.2	39.2	39.3	39.7	40.6	41.7

Note. VAI = value-added intensity. Headline totals are the official published totals. The 2023 observation reflects the Fifth National Economic Census benchmark; the 2018–2022 values retained here are the contemporaneous annual-release values, so the 2022–2023 comparison crosses a benchmark break. Sources: [23–29]; full source log in S1 Dataset.

### Service-oriented reallocation

The composition of value added moved toward services over the observed period (Table 2 and Fig 1). The service share rose from 64.8% in 2018 to 72.0% in 2024, reaching 72.7% in 2023 before easing by 0.7 percentage points in 2024. The manufacturing share fell from 33.7% to 26.5%, while facility construction remained between 1.5% and 2.0%. SMVAR rose from 1.92 to 2.72. The non-monotonic 2024 movement is important: the direction over the full period is clear, but the annual path does not support a claim of uninterrupted service expansion.

**Table 2:** Composition and category-specific value-added intensity, 2018–2024.

Measure	2018	2019	2020	2021	2022	2023	2024
Service VA share (%)	64.8	67.7	68.7	70.0	70.1	72.7	72.0
Mfg. VA share (%)	33.7	30.4	29.3	28.0	28.2	25.7	26.5
Facility VA share (%)	1.5	1.9	2.0	1.9	1.7	1.6	1.5
Service VAI (%)	51.3	51.0	52.2	51.7	51.6	51.5	52.1
Mfg. VAI (%)	25.7	25.1	25.6	25.3	25.9	26.1	27.8
SMVAR	1.92	2.23	2.35	2.50	2.49	2.83	2.72

Note. SMVAR = service-to-manufacturing value-added ratio; VAI = value-added intensity. Values are descriptive current-price accounting ratios. Sources: [23–29].

**Fig 1:**
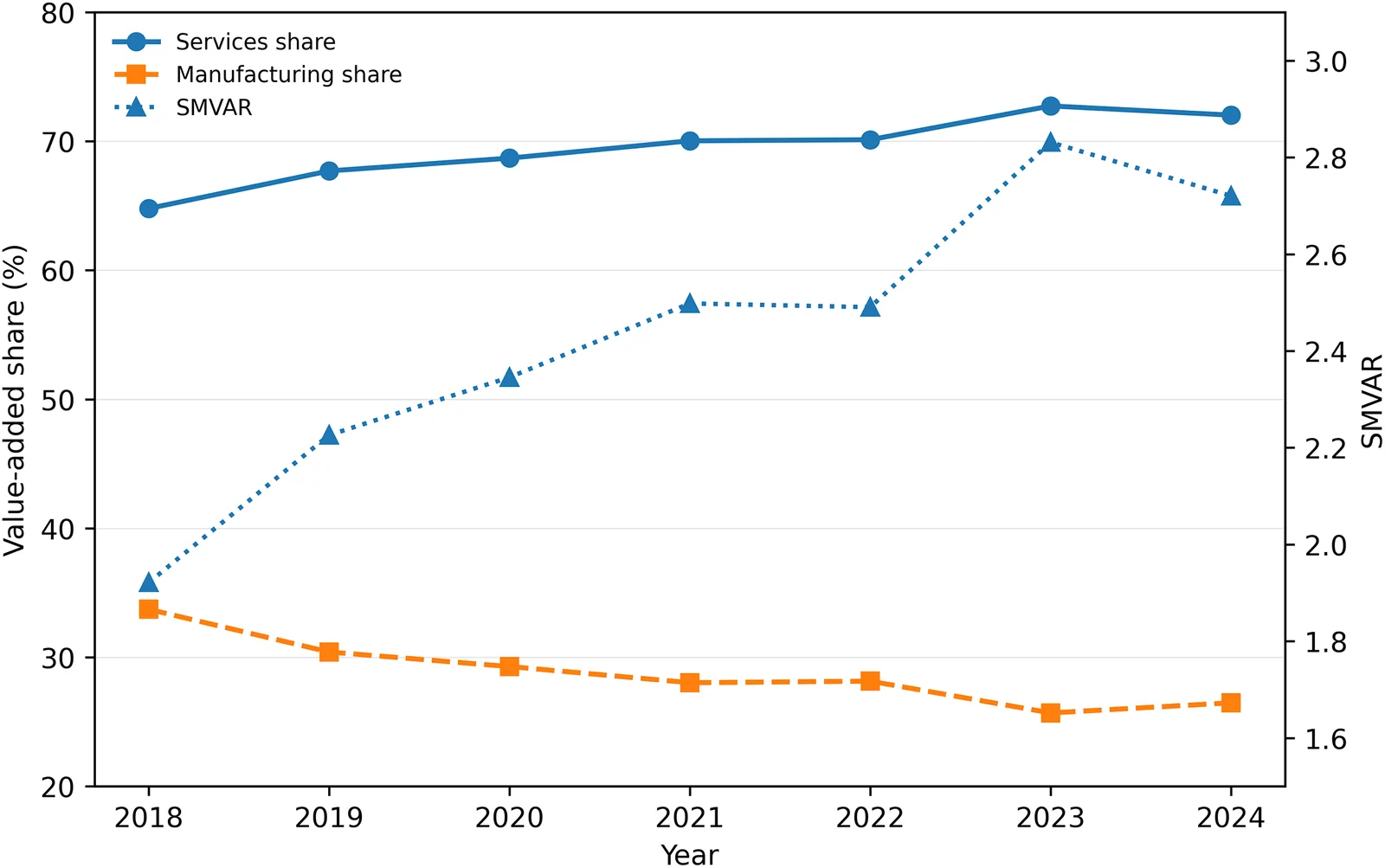
Value-added composition and service-to-manufacturing value-added ratio, 2018–2024. Service and manufacturing shares are shown on the left axis and SMVAR on the right axis. Values are based on current-price official accounts.

Category-specific intensities differed substantially. Sport services converted about 51–52% of gross output into value added throughout the period, compared with about 25–28% in manufacturing. Service VAI increased from 51.3% to 52.1%, and manufacturing VAI from 25.7% to 27.8%. These ratios indicate differences in intermediate-input structure and accounting value added per unit of gross output; they do not by themselves establish differences in economic efficiency or productivity.

### Shift-share decomposition and temporal robustness

For 2018–2024, aggregate VAI based on the published first-tier components increased by 3.81 percentage points. The between-category contribution was + 2.50 points, the within-category contribution +1.44 points, and the interaction −0.13 points (Table 3 and Fig 2). Thus, over the full window, changes in category gross-output weights contributed more to the aggregate increase than within-category intensity changes. The robustness analysis, however, qualifies any claim that this relationship was uniform over time.

**Table 3:** Shift-share decomposition across alternative multi-year windows.

Window	Between-category (pp)	Within-category (pp)	Interaction (pp)	Total change (pp)
2018–2022	+1.51	+0.22	+0.01	+1.74
2018–2023	+2.39	+0.29	−0.01	+2.67
2018–2024	+2.50	+1.44	−0.13	+3.81
2019–2024	+1.84	+1.84	−0.11	+3.58
2020–2024	+1.63	+1.01	−0.13	+2.51
2021–2024	+1.19	+1.34	−0.08	+2.45

Note. pp = percentage points. Decomposition uses the three published first-tier categories. Components may differ slightly from displayed totals because of rounding. Adjacent-year decompositions are reported in S1 Dataset.

**Fig 2:**
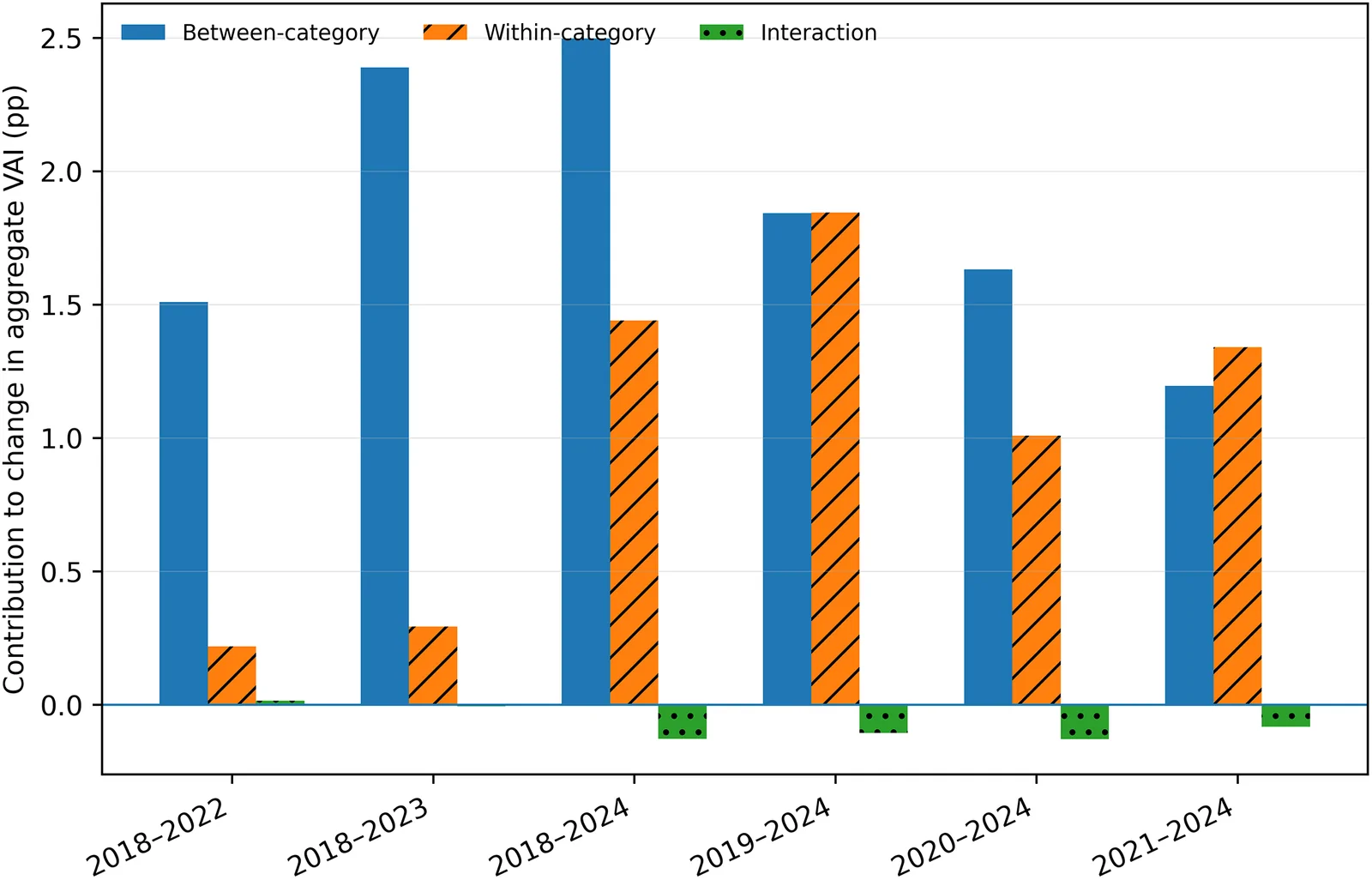
Shift-share contributions to the change in aggregate value-added intensity across alternative time windows. Positive values increase aggregate VAI; the interaction term may be negative. The figure emphasizes the 2018–2024 result and displays alternative windows as robustness checks.

The between-category contribution was positive in every multi-year window. It was clearly larger than the within-category component for 2018–2022, 2018–2023 and 2020–2024; the two were almost identical for 2019–2024, and the within-category contribution was slightly larger for 2021–2024. Adjacent-year results show the same temporal heterogeneity: the within component was negative in 2018–2019 and 2020–2021, while the between component remained positive in each adjacent interval. The evidence therefore supports a persistent positive reallocation contribution but not a single mechanism that dominates every subperiod.

### Sensitivity to differential price movements

Under the specified proxy-price scenario, the between-category contribution remained positive (Table 4). For 2018–2024, the between-category component was + 2.50 percentage points in current prices and +3.31 points under the proxy weighting scenario, while the within-category component remained +1.44 points by construction. When facility construction was excluded, the between-category contribution was + 2.59 points in current prices and +3.48 points under the proxy scenario. The proxy exercise therefore changes relative category weights rather than category-specific VAI.

**Table 4:** Proxy-price sensitivity of the 2018–2024 shift-share decomposition.

Specification	Between nominal (pp)	Between proxy (pp)	Within nominal (pp)	Within proxy (pp)	Total nominal (pp)	Total proxy (pp)
Three first-tier categories	+2.50	+3.31	+1.44	+1.44	+3.81	+4.57
Exclude facility construction	+2.59	+3.48	+1.46	+1.46	+3.92	+4.76

Note. Proxy results use the national CPI service-price index for services and the PPI for manufacture of cultural, educational, arts and crafts, sports and entertainment products for manufacturing. They are sensitivity estimates, not official constant-price accounts. Exact index values and formulas are in S1 Dataset.

In 2024, the proxy-weighted service share of first-tier value added was 74.65% compared with 72.03% in current prices, while SMVAR was 3.15 and 2.72, respectively. These scenario-specific differences reflect the selected relative-price proxies and should not be interpreted as evidence that inflation or relative-price effects have been definitively ruled out.

### Heterogeneous shock exposure and recovery

The 2020 contraction was heterogeneous within services (Table 5 and Fig 3). Under the primary classification, the three co-located categories together lost 16.24% of current-price value added between 2019 and 2020, retained 83.76% of their 2019 level, and then increased by 24.59% in 2021. Their combined 2021 value added was 4.35% above 2019, corresponding to a recovery ratio of 1.27. By contrast, education/training increased by 5.71% in 2020 and media/information by 18.91%, so neither had a 2020 recovery ratio because neither experienced a loss relative to 2019. Mixed services declined by 1.63% and manufacturing by 8.10% in 2020.

**Table 5:** Current-price shock and recovery indicators by exposure group, 2019–2021.

Exposure group	2020 vs 2019 (%)	2019 level retained in 2020 (%)	2021 vs 2020 (%)	2021 vs 2019 (%)	Recovery ratio
Co-located services	−16.24	83.76	+24.59	+4.35	1.27
Knowledge service	+5.71	105.71	+11.35	+17.71	n/a
Digital service	+18.91	118.91	+19.76	+42.41	n/a
Mixed services	−1.63	98.37	+14.46	+12.59	8.71
Manufacturing comparator	−8.10	91.90	+9.19	+0.35	1.04

Note. Recovery ratio = (VA2021 − VA2020)/(VA2019 − VA2020), defined only when 2020 value added is below the 2019 level. Values greater than 1 indicate that the 2021 current-price level exceeded the 2019 level. Because the denominator is the initial 2019–2020 loss, the recovery ratio can become very large when that loss is small. It should therefore be interpreted jointly with the 2020 percentage change, the 2021 rebound, and the 2021 level relative to 2019, rather than as a standalone ranking of resilience. Individual sub-sector metrics and classification sensitivity are in S1 Dataset.

**Fig 3:**
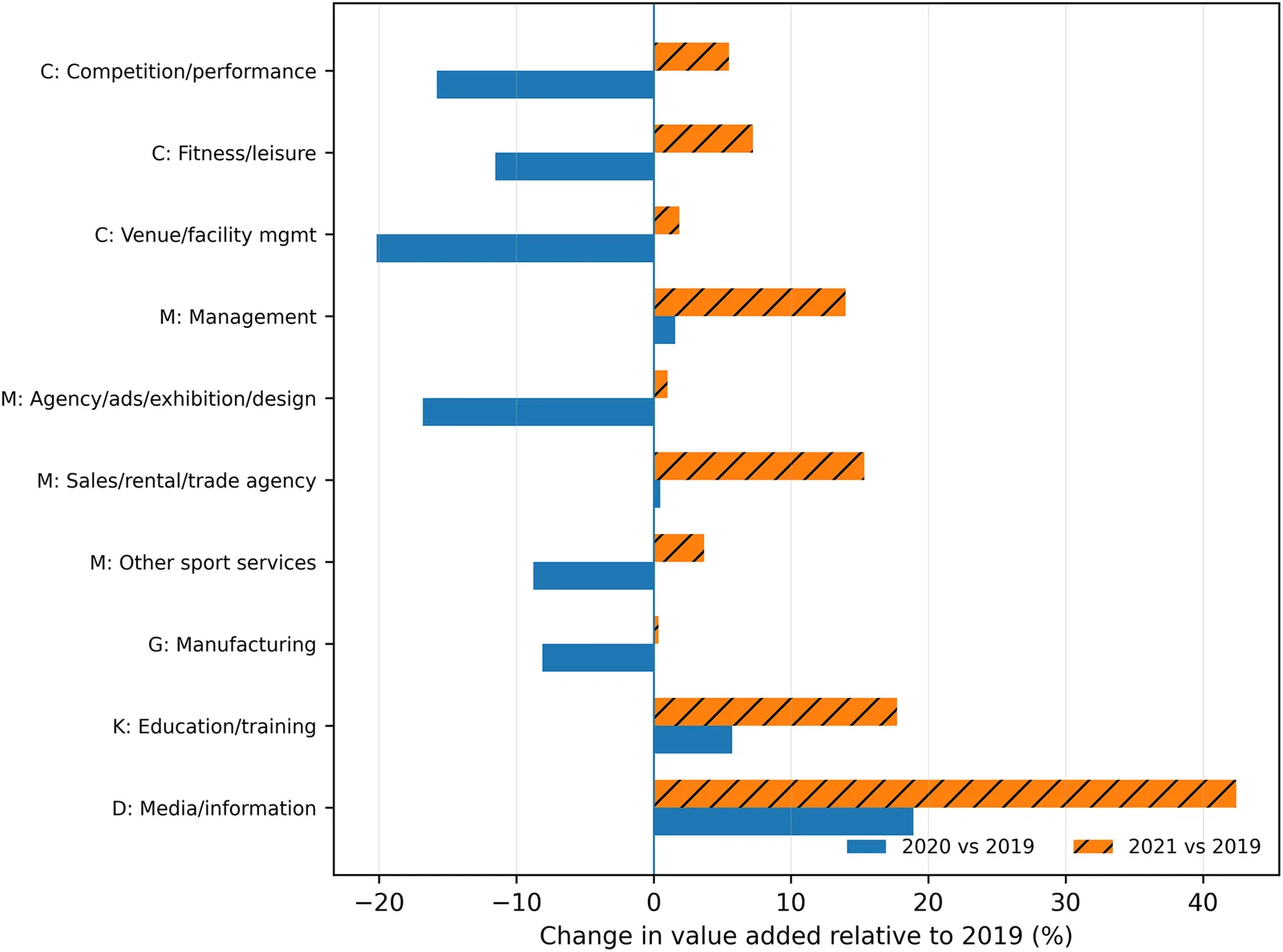
Shock and recovery of sport-industry sub-sectors relative to the 2019 baseline. The figure contrasts the 2020 percentage change from 2019 with the 2021 percentage difference from 2019. Exposure codes are C = co-located, M = mixed, G = goods, K = knowledge, and D = digital; values are current-price value added.

The recovery ratio of 8.71 for mixed services is driven by the group’s small initial 1.63% contraction in 2020 and should not be interpreted as evidence of proportionately greater resilience. The exposure contrast was not driven by the treatment of one borderline category. Reassigning agency/advertising/exhibition/design from mixed to co-located changed the combined co-located 2020 contraction only from −16.24% to approximately −16.27%. The results therefore describe greater observed short-run contraction among activities whose core delivery requires physical co-presence, while the aggregate data do not identify whether restrictions, demand changes, policy responses, prices or other contemporaneous mechanisms caused the differences.

## Discussion

The incremental contribution of this study is not the development of a new accounting method. Rather, it is the transparent and reproducible application of established accounting and shift-share techniques to an updated national sport-industry series, allowing between-category reallocation to be distinguished from within-category VAI change while testing temporal, classification, relative-price-proxy and shock-recovery sensitivity. Three findings emerge from the revised accounting analysis. First, the composition of China’s sport-industry value added moved toward services between 2018 and 2024, although the path was not monotonic. Second, the rise in aggregate VAI reflected both compositional reallocation and within-category changes. The between-category component was positive across all alternative multi-year windows, but it was not uniformly dominant, particularly in the post-2021 period. Third, the 2020 shock was highly uneven within services: categories centered on physical co-presence contracted sharply and then recovered, whereas education/training and media/information maintained positive current-price growth during 2020. These findings are more specific than a simple services-versus-goods contrast and more limited than a causal theory of structural transformation.

The full-period decomposition is consistent with the basic logic of structural reallocation. Services had substantially higher VAI than manufacturing, so movement in gross-output weights toward services mechanically raised aggregate VAI. This resembles the broader idea that sectoral composition matters for aggregate economic outcomes [7–10], but the short series cautions against describing the pattern as a completed or irreversible structural transformation. The 2024 partial reversal in value-added shares and the alternative-window results show that composition and within-category intensity can move differently from year to year. The 2019–2024 decomposition assigns almost equal contributions to between and within effects, while the 2021–2024 window assigns a slightly larger contribution to within-category change. The more defensible conclusion is therefore that service-oriented reallocation was one persistent positive contributor over the observed period, alongside time-varying within-category changes. Interpretation of the 2022–2023 movement requires additional caution because the 2023 release is census-benchmarked whereas the pre-2023 observations used here are contemporaneous annual-release values; part of that discontinuity may therefore reflect statistical rebenchmarking.

The interpretation of VAI also requires discipline. A higher VAI means that a larger share of gross output is recorded as value added after intermediate consumption; it does not necessarily mean that workers are more productive, firms are more profitable, or resources are used more efficiently. Service activities can have high VAI partly because they purchase fewer intermediate material inputs, while manufacturing can generate large gross output with substantial intermediate consumption. For the same reason, SMVAR is useful only as a compact description of the relative scale of service and manufacturing value added. The proxy-price exercise does not alter category-specific VAI because gross output and value added within each category are divided by the same proxy. Its role is therefore limited to testing the sensitivity of relative category weights and related aggregate measures under the specified proxies; it cannot rule out inflation or relative-price effects.

### Theoretical implications

The findings do not validate service-dominant logic or value co-creation at the macro level. Their theoretical implication is narrower: an observed rise in aggregate VAI can reflect both compositional reallocation and within-category change, so macro sport-industry trends should be decomposed before they are linked to theories of servitization or value creation [4–6,13–16]. In this sense, the study provides an accounting bridge between structural-reallocation research and sport-service scholarship while leaving the underlying organizational and consumer mechanisms to future micro-level research.

The crisis analysis adds an internal service distinction that is consistent with recent sport research on pandemic disruption and digital adaptation. Studies of sport networks and public events documented severe disruption to attendance-dependent activity [18,19], while research on sport entrepreneurship and digital transformation shows how technology supported adaptation during the pandemic [20,21]. In the present data, co-located categories contracted markedly in 2020 but exceeded their 2019 level by 2021, whereas media/information and education/training did not show a 2020 decline. This pattern is compatible with differential exposure to co-presence constraints, but the accounting design cannot isolate that channel from simultaneous differences in demand, government support, price movements, business closures or classification effects. Accordingly, the analysis is framed as heterogeneous exposure and recovery without assigning a causal effect to mobility restrictions or using evaluative performance language.

### Practical implications

The practical implication is to monitor the service–manufacturing mix, category-specific VAI, and exposure profiles separately rather than infer performance from aggregate VAI alone. The 2020–2021 contrast also supports differentiated contingency planning for venue-dependent activities and knowledge- or digitally delivered services. These implications concern monitoring and resilience planning; they do not establish that resources should be shifted from manufacturing to services or that any category offers superior returns. Manufacturing still accounted for more than one-quarter of sport-industry value added in 2024.

Cross-national transferability is possible only with substantial qualification. The United Kingdom and EU sport satellite accounts use definitions and estimation procedures designed for international comparability [1,2], while Japanese work demonstrates how an input–output framework can be tailored to a national sport-industry boundary [3]. China’s official classification is conceptually related but not identical: coverage, aggregation, price measures, data availability and benchmark revisions differ. The shift-share approach itself is portable because it requires only gross output and value added by compatible categories, but numerical comparisons of VAI or decomposition components across countries would require harmonized sport definitions and accounting treatment. The present study should therefore be read as a reproducible country case and a potential benchmarking approach, not as evidence that the same service transition occurs at the same rate elsewhere.

### Limitations and future research

The study has several limitations. First, seven annual observations are too short to establish a long-run structural regime, and the window contains both the COVID-19 shock and a late-period census benchmark revision. Because the 2018–2022 observations used here are contemporaneous annual-release values rather than a fully backward-revised census-benchmark series, decompositions spanning the 2022–2023 boundary should be interpreted as changes in the published accounting series, not as a pure measure of economic restructuring. The alternative-window and adjacent-year decompositions reduce dependence on a single endpoint but cannot create a longer historical series. Second, the official accounts are current-price measures. The proxy sensitivity analysis changes relative category weights but leaves category-specific VAI unchanged by construction; it therefore cannot substitute for sport-specific double-deflated volume measures or rule out price effects. Third, VAI is an accounting ratio shaped by intermediate-input conventions and should not be interpreted as productivity or profitability. Fourth, the co-location taxonomy is constructed from official category descriptions rather than firm-level operational data; sensitivity checks reduce but do not eliminate classification uncertainty. Fifth, aggregate national accounts cannot identify causal effects of policy, digitalization or pandemic restrictions, nor can they reveal firm heterogeneity or consumer-level value co-creation. Future research could extend the series as comparable post-census observations accumulate, construct deflated accounts if suitable price data become available, add employment and labor-productivity measures, compare harmonized sport satellite accounts across countries, and connect macro compositional changes to firm- and consumer-level service processes.

## Conclusion

In the published current-price accounts for 2018–2024, China’s sport industry exhibited a service-oriented reallocation: the service share increased from 64.8% to 72.0%, while the manufacturing share declined from 33.7% to 26.5%. Aggregate VAI rose from 37.9% to 41.7%. Shift-share decomposition indicates that changes in category output weights made a positive contribution to this rise across all tested multi-year windows, while within-category changes were also important and became relatively more prominent in some post-pandemic windows. Changes spanning 2022–2023 remain qualified by the Fifth National Economic Census benchmark break. Under the specified proxy-price scenario, the between-category contribution remained positive, but this sensitivity check concerns relative category weights and is not a full inflation-adjusted test of VAI. The 2020 shock further revealed substantial heterogeneity within services, with co-located activities experiencing a larger initial contraction than knowledge and digital service groups. These results characterize the accounting pattern observed over a short and disrupted period; they do not establish causal productivity gains or an irreversible long-run transformation.

## Supporting information

S1 Dataset Reproducibility workbook containing source logs, raw first-tier and sub-sector data, derived annual measures, classification rules, shift-share calculations, alternative-window and rolling robustness checks, price-proxy inputs and sensitivity analyses, crisis–recovery indicators, figure source data, and audit notes. (XLSX)

S2 File Archive containing S2_Code.py, README.txt and requirements.txt; the Python code reproduces the derived measures, robustness analyses, crisis–recovery calculations, and Figs 1–3 from S1 Dataset. (ZIP)
